# Plant biomarkers as early detection tools in stress management in food crops: a review

**DOI:** 10.1007/s00425-024-04333-1

**Published:** 2024-02-05

**Authors:** Omolola Aina, Olalekan O. Bakare, Adewale O. Fadaka, Marshall Keyster, Ashwil Klein

**Affiliations:** 1 Plant Omics Laboratory, Department of Biotechnology, University of the Western Cape, Robert Sobukwe Road, Bellville, 7530 South Africa; 2 Department of Biochemistry, Faculty of Basic Medical Sciences, Olabisi Onabanjo University, Sagamu, 121001 Nigeria; 3 Environmental Biotechnology Laboratory, Department of Biotechnology, University of the Western Cape, Robert Sobukwe Road, Bellville, 7530 South Africa

**Keywords:** Abscisic acid, Aquaporin, Dehydrin, Heat shock protein, Antioxidants, sRNA

## Abstract

**Main conclusion:**

Plant Biomarkers are objective indicators of a plant’s cellular state in response to abiotic and biotic stress factors. They can be explored in crop breeding and engineering to produce stress-tolerant crop species.

**Abstract:**

Global food production safely and sustainably remains a top priority to feed the ever-growing human population, expected to reach 10 billion by 2050. However, abiotic and biotic stress factors negatively impact food production systems, causing between 70 and 100% reduction in crop yield. Understanding the plant stress responses is critical for developing novel crops that can adapt better to various adverse environmental conditions. Using plant biomarkers as measurable indicators of a plant’s cellular response to external stimuli could serve as early warning signals to detect stresses before severe damage occurs. Plant biomarkers have received considerable attention in the last decade as pre-stress indicators for various economically important food crops. This review discusses some biomarkers associated with abiotic and biotic stress conditions and highlights their importance in developing stress-resilient crops. In addition, we highlighted some factors influencing the expression of biomarkers in crop plants under stress. The information presented in this review would educate plant researchers, breeders, and agronomists on the significance of plant biomarkers in stress biology research, which is essential for improving plant growth and yield toward sustainable food production.

## Introduction

Food is essential to our daily lives and well-being because it provides energy to power all metabolic processes and nutrients for proper growth and disease resistance (Holder 2019). Food security refers to the condition in which people always have social, economic, and physical access to safe, nutritious, and sufficient food to meet their dietary requirements for a healthy life (Sadati et al. 2021). Ensuring adequate food security for the global human population, projected to expand to 10 billion by 2050, a 34% increase over the current population size, is a paramount global concern and imperative (Boretti and Rosa 2019).

One of the foremost strategies to attain global food security entails a substantial boost in food crop production. A recent study conducted by Galieni et al. (2021) underscores the urgency of this matter, revealing that, given the current population growth rate, food production must surge by approximately 70% to align with existing food demand. Between 2000 and 2019, the total primary crop production recorded a 54% increase, reaching 9.4 billion tonnes (FAO 2022). However, this positive trend does not extend uniformly to developing countries. In stark contrast, food production per capita in Africa has experienced a decline of about 5–13% over the past few decades, with approximately 73 million people suffering from severe food insecurity (Mohamed et al. 2021; Bjornlund et al. 2020). The principal causes of global food insecurity are abiotic and biotic stress factors.

Abiotic factors such as drought, salinity, heavy metal stress, flooding, and extreme temperatures significantly impact crop production and contribute to food insecurity in developed and developing countries (Summy et al. 2020). These stress factors endanger approximately 90% of arable lands, leading to a 70% reduction in major food crops (Waqas et al. 2019). For instance, drought was the primary cause of grain production shortages in the twenty-first century, with approximately one-third of global drought incidents occurring in Sub-Saharan Africa. Ethiopia and Kenya, in particular, endured some of the most severe drought periods in the past four decades (Kogan et al. 2019; Ofori et al. 2021). Furthermore, global temperature will rise by 2–4.9 °C by 2100, and approximately 5 million sites will experience heavy metal contamination at concentrations above regulatory limits (Raftery et al. 2017; Gonzalez Henao and Ghneim-Herrera 2021).

Biotic stress factors affect crop production and food security worldwide (Kaur et al. 2021). These factors, including bacteria, viruses, fungi, nematodes, weeds, and insects, are a huge constraint, destroying about one-third of agricultural produce valued at 750 billion US dollars annually (Mesterházy et al. 2020). According to the Food and Agriculture Organization (FAO) of the United Nations (UN), plant diseases alone incur global damages of 220 billion USD, while uncontrolled weeds could cause a 100% loss in crop yield annually in both developing and developed nations (He and Krainer 2020; Chauhan 2020). Biotic stress factors have historically played a role in some of the most severe famines. For example, in the United States, *Puccinia graminis tritici* fungi caused an epidemic that resulted in the loss of millions of bushels of wheat (Prasad et al. 2023). Additionally, the cassava mosaic disease epidemic in India, Sri Lanka, and Kenya has resulted in a yearly loss of approximately 25 million tons of cassava, which can lead to famine in subsequent years, especially in countries where it is a staple crop. These multifaceted challenges pose a significant threat to food security on a global scale.

Developing innovative methods and technologies to control or enhance plants' resistance to stress factors has become critical in improving crop growth and yield (Hareesh et al. 2023). An integral component of advancing these methodologies is gaining a profound understanding of plant response patterns to external influences (Galieni et al. 2021). Plants have a dynamic homeostasis system, enabling them to maintain a stable internal state, even amidst unpredictable external conditions. This equilibrium is crucial for their survival and optimal functionality (Torday 2015).

Plants synthesize biomarkers in response to stress to regulate cellular homeostasis. These biomarkers represent specific molecules or compounds that serve as measurable and quantifiable indicators of a plant's reaction to external stimuli (Steinfath et al. 2010). A diverse array of substances, including phytohormones, enzymes, proteins, and nucleic acids, constitute plant biomarkers, serving a pivotal role in monitoring and responding to changes in a plant's environment. Furthermore, they function as precursors, enabling the detection of potential stress well before it manifests as physical symptoms (Alharbi 2020).

Understanding plant physiology and developing strategies to improve crop resilience and productivity in changing environmental conditions. Studying plant biomarkers is an essential aspect of achieving this goal (Zhou et al. 2022). This review presents an overview of the typical cellular biomarkers expressed by plants in response to abiotic and biotic stress factors. It also discusses methods of identifying these biomarkers, their importance in crop engineering, and factors influencing their expression. The information provided in this review would enable agronomists and plant biotechnologists to develop rapid intervention mechanisms to improve crop resilience against both abiotic and biotic stress factors, thus contributing to global food security.

## Plant biomarkers

Biomarkers have played a role in modern science for over half a century, but their significance has seen a noticeable increase since the twenty-first century. This surge can be attributed to new technological advancements that have made it possible to generate and validate biomarkers (Rapley and Whitehouse 2015). Biomarkers are indicators of the cellular state of an organism in response to environmental and biological factors (Paniagua-Michel and Olmos-Soto 2016). They are quantifiable and reproducible, and their concentrations differ significantly from those found in normal, unaffected organisms (Bodaghi et al. 2023). Plants, for instance, synthesize biomarkers in response to abiotic and biotic stress factors. These biomarkers function as early warning signals in plants, allowing the detection of stressors before they cause severe damage, often manifested as physical symptoms (Ernst 1999).

There are numerous laboratory-based techniques available for detecting and analyzing biomarkers in plant tissues. These methods involve examining either the biomarker itself or the genes that encode it (Pérez-Clemente et al. 2013). Examples of these techniques include Western blotting, MALDI-TOF, SDS-PAGE, 2D-GE, northern blotting, enzyme-linked immunosorbent assay (ELISA), LC–MS, and polymerase chain reaction (PCR) (Yang et al. 2021). In recent years, omic technologies have provided a more holistic understanding and aided in identifying plant biomarkers indicative of stress conditions. These include techniques such as genomics to identify significant stress-associated genes, proteomics to study variations in protein abundance relative to induced stress, metabolomics to study variations in cellular metabolites in response to stress, and transcriptomics to analyze gene expression patterns (Roychowdhury et al. 2023). Furthermore, the advancement of next-generation sequence approaches such as microarrays, RNA sequencing, and single-molecule real-time sequencing have provided high-throughput, sensitive and rapid methods of generating data from omic techniques (Saeed et al. 2022; Udawat 2023).

While plant biomarkers may not exhibit the same level of specificity as those in mammalian systems, they still play a significant role in detecting and mitigating plant stress factors (Steinfath et al. 2010). Given the increasing impact of abiotic and biotic stressors on plants, there is a growing global interest in using biomarkers at cellular and molecular levels to detect stress early, monitor changes in plant metabolism in response to stress, and prevent irreversible damage (Fernandez et al. 2016; Paes de Melo et al. 2022). This review will explore plant biomarkers with differential expression patterns under stress conditions. These biomarkers include abscisic acid, aquaporin, dehydrin, transcription factors, heat shock proteins, antioxidant enzymes, and sRNA.

## Abscisic acid as a hormonal biomarker in plant stress responses

**Fig. 1:**
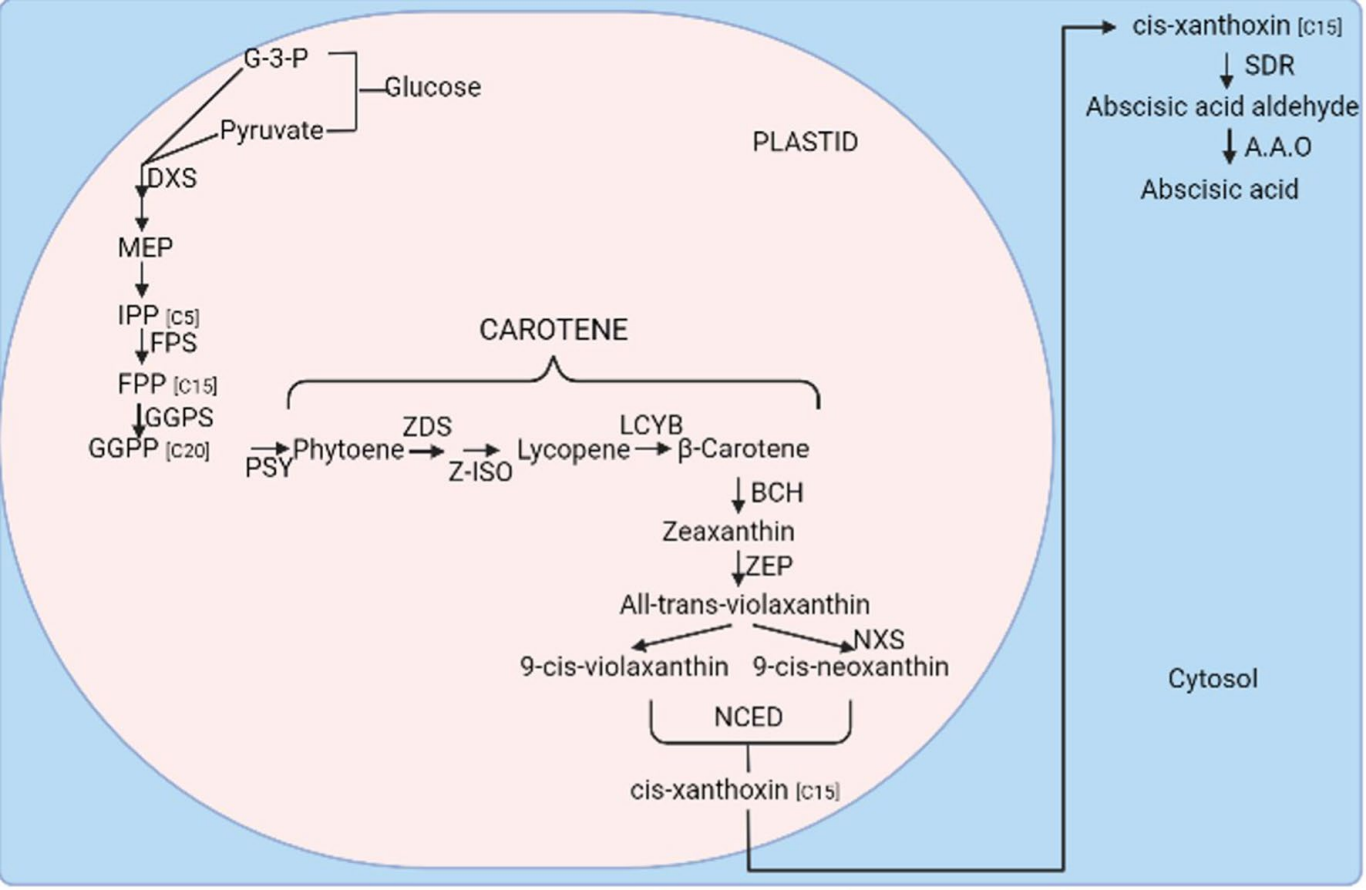
The biosynthesis of Abscisic acid via the direct and indirect pathway. G-3-P—glyceraldehyde-3-phosphate; DXS—Deoxyoxylulose-5-phosphate synthase; MEP-2-C-methyl-d-erythriotl-4-phosphate; IPP—Isopentenyldiphosphate; FPS—Farnesyl diphosphate synthase; FPP—Farnesyl pyrophosphate; GGPS—geranylgeranyl pyrophosphate synthase; GGPP—Geranylgeranyl pyrophosphate; PSY—Phytoene Synthase; ZDS—phytoene desaturase; Z—ISO-z-carotene desaturase; LCYB—lycopene cyclase; BCH—β-carotenoid hydroxylase; ZEP—Zeaxanthin epoxidase; NXS—Neoxanthin synthase; NCED—9-cis-epoxycarotenoid dioxygenase; SDR—Short chain dehydrogenase; A.A.O—Abscisic acid oxidase

Abscisic acid (ABA) is a sesquiterpenoid with 15 carbon atoms synthesized from β-carotene via either the carotenoid pathway or the indirect pathway (mevalonic acid-independent pathway), as demonstrated in Fig. 1 (Hewage et al. 2020; Chen et al. 2020).

ABA plays a crucial role in the growth and development of plants, acting as an essential phytohormone (Chen et al. 2020). It is especially important in regulating several biochemical, molecular, and physiological processes in crop plants that are exposed to harsh abiotic stress conditions such as drought, salinity, extreme temperature, UV—radiation, and heavy metal stress (Vishwakarma et al. 2017). ABA is a key factor in synchronizing multiple processes that confer stress tolerance, such as root cell elongation, stomata closure, activating transcriptional and post-transcriptional stress defense responses, inducing the expression of stress-related genes, increasing hydraulic conductivity, and metabolic alterations (Muhammad Aslam et al. 2022). Numerous studies have found that an increase in cellular ABA concentrations is linked to adverse environmental conditions (Wang et al. 2021; Hu et al. 2022b; Yang et al. 2023b). For instance, under salt stress, rice plants accumulate ABA, which affects the growth and development of the root meristem (Huang et al. 2021). Similar examples of changes in the cellular concentration of ABA in crop plants under various abiotic stress conditions and their mode of action are reported in Table 1.

**Table 1:** Abscisic acid as a hormonal biomarker in plants' adaptive response to various Abiotic stress

Stress	Plant	Analytical Technique	Mechanisms/process	References
Salinity	*Vitis vinifera* L.	liquid chromatography-mass spectrometry	An increase in endogenous ABA in the leaves enhanced stress tolerance by increasing osmotic stress tolerance and water regulation	Ekinci et al. (2023)
Heavy metal stress (Cadmium, zinc, nickel and copper)	*Zea mays* L.	Gas chromatography-mass spectrometry	Increased concentration of ABA in the leaves and roots confers tolerance by activating defense mechanisms such as stimulating stomata closure, reducing oxidative stress, and reducing the uptake of heavy metals by the root	AbdElgawad et al. (2020)
Heat	*Zea mays* L.	High-performance liquid chromatography-mass spectrometry	Accumulation of ABA in the leaves enhanced tolerance to heat stress by activating the ABA-mediated defense pathway	Sun et al. (2023)
Cold	*Solanum lycopersicum* L.	High-performance liquid chromatography-mass spectrometry	An increase in ABA concentration induces the jasmonic acid signalling pathway, activating the CBF pathway and enhancing cold stress tolerance	Wang et al. (2016)
Drought	*Phaseolus vulgaris* L.	Enzyme-linked immunosorbent assay (ELISA)	Accumulation of ABA in the leaves enhances resilience against drought by regulating stomata closure and gene expression	Wang et al. (2019a)
Drought	*Brassica napus* cv. Capitol	HPLC–ESI–MS	Accumulation of ABA increases resilience by stimulating the degradation of chlorophyll, leaf senescence, degradation of starch, and expression of sucrose transporter genes	Park et al. (2021)

In contrast to abiotic stress, the accumulation of ABA during biotic stress can either increase plant tolerance or susceptibility (Gietler et al. 2020). The impact of ABA on plants is also influenced by pathogen type and biotic stress conditions (Bharath et al. 2021; Rasool 2022). High levels of cellular ABA can suppress the immune response of crop plants by repressing salicylic acid activity, making them more susceptible to biotrophic pathogens, such as *Magnaporthe oryzae* (in barley) and *Botrytis cinerea* (in tomato), which infect living host cells (Ulferts et al. 2015; Sivakumaran et al. 2016). On the other hand, the accumulation of ABA in crop plants under biotic stress causes stomata closure and increases callose deposits, which protects them from pathogen invasion (Hewage et al. 2020). Table 2 highlights additional examples of changes in cellular ABA concentration in response to biotic stress, including their mechanism of action.

**Table 2:** Abscisic acid as a hormonal biomarker in plants’ adaptive response to various biotic stress

Stress	Plant	Analytical technique	Mechanisms/process	References
Fungi (*Fusarium oxysporum* f.sp. lini)	*Linum usitatissimum* L.	ABA Immunoassay kit	Accumulation of ABA inhibits pathogen invasion by stimulating stomata closure, inducing expression of defense genes, and facilitating callose deposition on plant cell walls	Boba et al. (2020)
*Fungi (Botrytis cinerea)*	*Vitis vinifera* L.	High-performance liquid chromatography-mass spectrometry	An increased cellular concentration of ABA increases the activity of cell wall-loosening genes, resulting in increased susceptibility to pathogen attack	Coelho et al. (2019)
Rice stripe virus	*Oryza sativa* L.	High-performance liquid chromatography-mass spectrometry	Accumulation of ABA within infected rice shoots represses ferredoxin expression, consequently compromising the plants' immune response and making them more susceptible to viral attack	Cui et al. (2021)
Potato virus Y	*Solanum tuberosum* L.	High-performance liquid chromatography-mass spectrometry	Elevated levels of ABA enhance callus synthesis within the plasmodesmata and suppress the activity of the callose-degrading enzyme β-1,3-glucanase, thus preventing the intrusion of viruses into host plant cells."	Al-Mokadem et al. (2022)
Nematode (*Heterodera avenae* Woll*.*)	*Triticum aestivum* L.	High-performance liquid chromatography mass spectrometry	"The accumulation of ABA in infected plant tissues enhances resistance to nematode attacks by acting as a signalling hormone that triggers the activation of the systemic defense pathway in the host plant."	Korayem et al. (2022)
Nematode (*Meloidogyne incognita*)	*Solanum lycopersicum* L.	liquid chromatography-mass spectrometry	"Reduced cellular ABA levels stimulate strigolactone synthesis, which increases defense against nematode attacks	Xu et al. (2019)
Aphid (*Melanaphis sacchari* Zehntner)	*Sorghum bicolor* L.	liquid chromatography-mass spectrometry	An increased cellular concentration of ABA triggers crosstalk between the JA and ABA-mediated defense pathways, enhancing resistance against insect attacks	Huang et al. (2022)
Insect (*Aphis glycines* Matsumura)	*Glycine max* L.	liquid chromatography-mass spectrometry	Increased endogenous ABA level stimulates the activation of the JA-induced defense pathway, resulting in improved tolerance to soybean aphid attacks	Chapman et al. (2018)

## Aquaporin as a biochemical marker in plant stress responses

Aquaporins (AQP) are transmembrane proteins that range in molecular weights from 23 to 31 kDa (Kapilan et al. 2018). They are found in various parts of plants, such as roots, leaves, seeds, flowers, and fruits, under normal physiological and stressful conditions (Hoai et al. 2020; Li et al. 2022)). Given the sedentary nature of plants, their numerous intracellular compartments, and the lack of a specialized circulatory system, there is a critical need for coordinated water regulation to adapt to multiple abiotic stresses, including salinity, drought, temperature, nutrient limitation, and heavy-metal toxicity (Banerjee and Roychoudhury 2020).

AQP serves as a channel for transporting and maintaining cellular water, ion, and neutral solutes, which explains their vital role in regulating some physiological and metabolic processes, such as root/leaf hydraulic conductivity, cell osmoregulation, transpiration, stomatal closure, cell regeneration, and cell elongation in plants (Zupin et al. 2017). Given the importance of AQP in plant cells, they are either upregulated or downregulated in response to stress. This modification of AQP abundance under different stressors helps to regulate osmotic balance (Kapilan et al. 2018).

AQP are typically classified into five subfamilies, namely tonoplast intrinsic protein (TIP), plasma membrane intrinsic proteins (PIP), small basic intrinsic proteins (SIP), X intrinsic proteins (XIP), and nodulin 26-like intrinsic proteins (NIP) (Zargar et al. 2017). These families of proteins perform specific roles within the cell during normal physiological conditions. The SIPs and some NIPs mediate the transportation of solvents within the endoplasmic reticulum, and the TIPs and NIPs mainly participate in the movement of minerals and organic micro-compounds due to their lower permeability to water molecules (Afzal et al. 2016). Conversely, the PIPs and TIPs mainly play a part in reactions during drought, cold, and salinity stress (Maurel et al. 2015).

Notably, plants’ resistance to different stressors is directly proportional to the amount, distribution, and efficiency of aquaporins within the cells (Patel and Mishra 2021). For example, a study by (Lian et al. 2006), showed that 20% polyethene glycol (PEG)-induced water stress enhanced the accumulation of root and leaf plasma membrane intrinsic proteins in two rice cultivars, lowland rice (*Oryza sativa* L. cv. Xiushui 63) and upland rice (*Oryza sativa* L. cv. Zhonghan 3). Under drought stress, there was a consistent increase in the expression of the aquaporin gene (PIP1;5) in the leaves and roots of the more drought-tolerant pearl millet (*Pennisetum glaucum* (L.) R. Br) (Iwuala et al. 2020). Further examples of aquaporin expression under different abiotic stress conditions and their mode of action are shown in Table 3.

**Table 3:** Aquaporin, a biochemical marker in plants' adaptive response to various abiotic stress

Stress	Plant	Analytical Technique	Family/Loci	Mechanisms/process	References
Heavy metal stress (Arsenic)	G*lycine max* L.	RNA-seq and RT-PCR	Not specified	Accumulation of AQP enhanced stress tolerance by facilitating the uptake of arsenic ions by the root and phytochelatin or conjugation by glutathione	Zeeshan et al. (2023)
Salinity	*Phaseolus vulgaris* L.	Western blot	Family: PIP2;1 Locus: AGV54658 Family: PIP2;2 Locus: ABU94630	Redistribution and accumulation of AQP in the root cortex increases resilience against salt stress	Calvo-Polanco et al. (2014)
Cold	*Oryza sativa* L.	RNA-seq, qRT-PCR SDS -PAGE and Western blot	Family: PIP 1;1 LOC4330248 Family:PIP2;1 LOC4343122	Downregulation of AQP proteins and decreased gene expression in the roots and shoots of the rice cultivars enhanced tolerance to cold by regulating water translocation within the tissues	Yu et al. (2006)
Drought	*Vitis vinifera* L.	qRT-PCR	Family:PIP2;1 Locus: AGV54658	Accumulating AQP protein in the roots facilitates water transportation into the cells, reducing the adverse effect of increased transpiration	Koc et al. (2017)
Salinity	*Camelina sativa* L.	SDS-PAGE and Western blot	Family: PIP2;1 LOC104791606	A decrease in the tissue’s expression of aquaporin genes lowers water conductance, thereby enhancing stress tolerance	Kim et al. (2019)
Drought	*Solanum lycopersicum* L.	1-D Electrophoresis and Immunoblotting	Family: PIP1;3 Locus: PIP1-3 Family: PIP1;4 Locus: PIP1-4	Aquaporin accumulation in leaves promotes effective water transport within plant cells and maintains hydraulic conductivity	Conti et al. (2022)
Heat	*Glycine max* L.	RT-PCR	Family: PIP1;1 Locus: PIP1-1	Differential expression of aquaporin in soybean tissues enhanced heat tolerance by regulating thermos-tolerance	Feng et al. (2019)
Drought	*Oryza sativa* L. cv. Xiushui 63) and (*Oryza sativa* L. cv. Zhonghan 3)	Western blot and RT-PCR	Family:PIP1;2 LOC9270874 Family: PIP1;3 LOC4331194 Family: OsPIP2;1 LOC4343122	The upregulation of the AQP gene and its corresponding protein in leaves and roots enhances water permeability and movement across the cellular membrane	Lian et al. (2006)

## Dehydrin as a biochemical marker in plant stress responses

Dehydrins (dehydration-induced proteins) are a type of protein that are highly hydrophilic and thermostable, with molecular weights ranging from 22 to 60 kDa (Arumingtyas and Savitri 2013). These proteins belong to group 2 within the late embryogenesis abundant (LEA) family and are the most extensively studied of the seven groups of LEA proteins due to their crucial role in increasing plant tolerance to abiotic stress (Mertens et al. 2018).

During times of abiotic stress, plant organs accumulate dehydrins within their nucleus, mitochondria, cytoplasm, and membranes (Tiwari and Chakrabarty 2021). Due to their hydrophilic and thermostable properties, these proteins are able to maintain structural flexibility, even binding to membrane proteins during water deficit to prevent protein inactivation and coagulation (Liu et al. 2017). Furthermore, dehydrins’ highly disordered and unstructured nature plays a crucial role in increasing tolerance to abiotic stress by preserving cellular integrity through the formation of hydrogen bonds within the cell membrane via coupled folding (Banerjee and Roychoudhury 2016).

According to (Kalemba et al. 2015), dehydrin accumulation in various cellular compartments, organelles, and membranes in beech (*Fagus sylvatica* L.) seeds during development and storage prevents cellular damage caused by dehydration. Table 4 shows other examples of dehydrin expression in different crop plants under various abiotic stress conditions.

**Table 4:** Dehydrin, a biochemical marker in plants' adaptive response to various abiotic stress

Stress	Plant	Analytical Technique	Family/Loci	Mechanisms/process	References
Drought	*Glycine max* L. Merr	SDS PAGE and Western blot	Family: SK3 (DHN 1) LOC100816147	Accumulation of dehydrin proteins increases the resilience of the drought-tolerant cultivar by facilitating membrane stability, ion flow, and water retention	Arumingtyas and Savitri (2013)
Cold, drought and salinity	*Triticum aestivum* L.	RNA-seq	Family: SK3 (DHN7) LOCUS: AF7085145	Overexpression of the SK3-type dehydrin gene (TaDHN7) improves stress tolerance by stabilizing cellular structures and macromolecules	Hao et al. (2022)
Drought	*Coix lacryma-jobi* L.	RNA-seq and qRT-PCR	Family: DHN1 LOC100816147	Upregulation of the dehydrin gene improves tolerance by mitigating oxidative damage	Miao et al. (2021)
Drought	*Cucumis melo* L.	Western blot	Not specified	Accumulation of dehydrin proteins during water stress prevents the denaturing of macromolecules and maintains turgor pressure	Motallebi-Azar et al. (2019)
Salinity	*Hordeum vulgare* L.	SDS-PAGE and Immunoblot assay	Family: K (DHN 5) LOCUS: AAD02262	Accumulation of dehydrin protein confers tolerance via its radical scavenging, cryoprotective, ion binding, and chaperone function in the cell	Kosová et al. (2015)
Cold and drought	*Solanum sogarandinum* L.	Western blot	Family: SK3 (DHN24) LOCUS: AAP44575	The accumulation of DHN 24 in the roots, stems, and leaves stabilizes macromolecules by facilitating the formation of intermolecular hydrogen bonds, hence increasing stress tolerance	Szabala et al. (2014)
Salinity	*Triticum aestivum* L.	LC–MS	Not specified	Increased expression of dehydrin proteins improves adaptation to salt stress by efficiently regulating ion balance, osmotic pressure, oxidative stress, and protein damage	Khan et al. (2023)

Dehydrins are grouped structurally into five sub-classes, namely SKn, Kn, YnKn, KnSYn, and YnSKn, based on the presence of conserved sequences (lysine-rich K-segment, unique to all Dehydrins; serine-rich S-segment; and tyrosine-rich Y-segment (Sun et al. 2021b). These conserved regions play essential roles in protecting plants from the adverse effects of osmotic stress. For example, the K-segment binds to cell membrane proteins, protecting them from electrolyte leakage and lipid oxidation. The S-segment is responsible for phosphorylation by the SNF1-related protein kinase, which influences the translocation of dehydrins from the cytosol to the nucleus and binding to calcium ions. However, the precise function of the Y-segment remains unknown (Murray and Graether 2022). Stival and colleagues reported the expression of dehydrin genes in *Picea glauca* in response to drought. They discovered that dehydrins with N1 K2 and N1 AESK2 sequences were the most receptive to the absence of water (Stival Sena et al. 2018).

## Transcription Factors as molecular biomarkers in plant stress responses

As stated by Kabir et al. (2021), transcription factors (TFs) are multifunctional proteins that regulate various plant reactions to stress. They bind to transcription-factor binding sites in the promoter region of a DNA sequence, which then triggers a series of downstream reactions that result in the expression of target genes and the subsequent synthesis of functional proteins relative to stress (Wu et al. 2015). Stress signals perceived by cell wall and membrane receptors are transmitted to transcription factors through intracellular compounds such as reactive oxygen species (ROS), Ca^2+^, phosphatases and protein kinases. These TFs then regulate or stimulate the expression of responsive genes by binding to their respective *cis*-element (Shahzad et al. 2021).

Numerous families of transcription factors highly regulate plant defense gene expression in response to abiotic and biotic stress factors. These families include basic leucine zipper (bZIP), AP2/ERF, WRKY, NAC (NAM: no apical meristem, ATAF, CUC: cup-shaped cotyledon), drought-response elements binding proteins (DREB) and myeloblastoma (MYB) (Javed et al. 2020; Hrmova and Hussain 2021). Each of these transcription factor families comprises over 100 and can act as positive or negative regulators to enhance tolerance to the respective stress factor (Hu et al. 2022c).

Several research have reported changes in the expression of TF in crop plants in relation to abiotic stress. For example, (Xiang et al. 2008) reported an increased expression of a member of the bZIP transcription factor family (OsbZIP23) in drought-resistant upland rice genotype IRAT109 (Japonica) exposed to drought and salinity stress, as revealed through Northern-blot analysis. (Rahman et al. 2016) reported that the overexpression of finger millet (*Eleusine coracana* L.) transcription factor (NAC 67) in rice increased the tolerance of the resulting transgenic rice to drought stress by increasing the relative water content. Similarly, (Wei et al. 2019) demonstrated that overexpressing the GmWRKY54 transcription factor in soybeans conferred drought tolerance by activating target genes in the Ca^2+^ and abscisic acid signalling pathway. Other examples of TF expression under different abiotic stress conditions and their mode of action are shown in Table 5.

**Table 5:** Transcription factor as a biochemical marker in plants' adaptive response to various abiotic stress

Stress	Plant	Analytical Technique	Family/Loci	Mechanisms/process	References
Salinity	*Triticum aestivum* L.	qRT-PCR	Family: LOC100873097MYB3 Family: MYB4 LOC123099635 Family: MYB13 LOC123181226 Family: MYB59 LOC123063737	Increased TF expression stimulates the expression of other genes, including 2-oxoglutarate-dependent dioxygenase, which improves salinity stress tolerance	Sukumaran et al. (2023)
Drought	*Hordeum vulgare* L.	DAP-seq and qRT-PCR	Family: NAC LOC123430376 Family: MYB LOC123426548 Family: bZIP LOC123398640 Family: AP2/ERF-ERF LOC123444167	Upregulation of TF increases resistance to salinity stress by activating the synthesis of polyphenols via the phenylpropanoid pathway	Wang et al. (2023b)
Drought	*Citrus sinensis* L.	qRT-PCR	Family: AP2/ERF Locus: MYC2_ARATH	Overexpression of the AP2/ERF TF in the leaves confers tolerance by regulating the expression of numerous drought-responsive genes	Ito et al. (2015)
Drought and Salinity	*Oryza sativa* L.	RNA-seq and qRT-PCR	Family: AP2/EREBP LOC4345697 Family: MYB Locus: LOC4346661 Family: bHLH Locus: LOC4343984 Family: NAC Locus: LOC4334553	Upregulation of various TF confers tolerance by increasing the expression of genes that regulate ABA and JA-mediated stress response pathways	Huang et al. (2014)
Cold	*Oryza sativa* L.	qRT-PCR	Family: bZIP LOC4325061	Upregulation of the ABF1 TF improves tolerance to cold stress by increasing trehalose accumulation and proline synthesis and decreasing electrolyte leakage	Shu et al. (2023)
Heavy metal stress (Arsenic)	*Oryza sativa* L.	qRT-PCR	Family: NAC3 LOC4342753	Over expression of SNAC3 TF in increased stress tolerance by regulating stress-related gene expression, osmolyte accumulation and activity of antioxidant enzymes	Pooam et al. (2023)

TFs are also crucial in the plants’ adaptation mechanism to biotic stress. For example, during a pathogenic attack, TF promotes the activation of pathogenesis-related protein genes and hypersensitive response, thereby increasing the plants’ resilience to the pathogen (Campos et al. 2022). (Kaushal et al. 2021) also reported the upregulation of NAC, bHLH, and MYB transcription factors in banana cultivars resistant to *Fusarium* stress. Similarly, López et al. (2021) observed an increased resistance of a Columbia tomato cultivar to *Fusarium oxysporum* f. sp. *lycopersici* (Fol) infection due to the upregulation of WRKY transcription factor. Table 6 highlights more examples of TF expression under biotic stress.

**Table 6:** Transcription factor as a biochemical marker in plants' adaptive response to various biotic stress

Stress	Plant	Analytical Technique	Family/loci	Mechanisms/process	References
Fungi (*Rhizoctonia solani*)	*Zea mays* L.	RNA-seq	Family: NAC41 Locus: PWZ57351	Increased NAC41 TF expression improves resistance to fungal invasion by activating the SA-mediated defense mechanism	Cao et al. (2022)
Wounding	*Oryza sativa* cv. Nipponbare	Northern blot and qRT-PCR	Family: NAC6 Locus: AEO53058	Upregulation of TF increases tolerance to wounding by facilitating quick response to the biotic stimuli	Ohnishi et al. (2005)
Nematode (*Globodera rostochiensis*)	*Solanum tuberosum* L.	RNA-seq and qRT-PCR	Family: WRKY LOC102596618	Upregulation of WRKY TF improves tolerance to nematode attack by preventing ROS-induced cell death	Bairwa et al. (2023)
Insect (*Nilaparvata lugens*)	*Oryza sativa* L.	RT-qPCR	Family: MYC2 LOC4349484	MYC2 transcription factor expression enhances resistance against insects by activating the JA defense pathway via promoting the synthesis of mixed-linkage β-1,3;1,4-d-glucan (MLG), which, in turn, reinforces vascular wall thickness and activates the LECTIN RECEPTOR KINASE1–mediated (OsLecRK1) defense signalling	Dai et al. (2023)
Insect (*Aphis glycines Matsumura*)	*Glycine max* L.	RNA-seq and qRT-PCR	Family: AP2/ERF LOC102660503 Family: WRKY LOC100782726 Family: MYB Locus: MYB118	The induction of TF enhances resistance against aphid invasion by regulating crosstalk between JA and SA defense pathways and promoting callose deposition at insect-feeding sites	Yao et al. (2020)
Grapevine berry inner necrosis virus (GINV)	*Vitis vinifera *L.	qRT-PCR	Family: MYB LOC100254518	Upregulation of the MYB TF enhances resistance by suppressing the growth of the virus	Wang et al. (2023a)

## Heat shock proteins as a biochemical biomarker in plant stress responses

Heat shock proteins (HSP), commonly known as stress proteins, are expressed by all living organisms, including plants and are widely distributed in cellular compartments and organelles such as the nucleus, endoplasmic reticulum, cytoplasm, and chloroplast (ul Haq et al. 2019; Singh et al. 2019). These proteins can be classified into five families based on their sequence homology and molecular weight: small Hsps (sHsp), Hsp60, Hsp70, Hsp90, and Hsp100 (Li and Liu 2019). Under normal physiological conditions, HSP constitutes between 5 and 10% of the total concentration of cellular proteins, where they play a highly significant role in regulating various growth and developmental processes such as controlling the cell cycle, assembling multi-protein units transporting into and out of subcellular compartments, and controlling protein degradation (Park and Seo 2015). However, their expression significantly increases under abiotic and biotic stress, a crucial adaptation to crop plants' stress tolerance (Hu et al. 2022a).

Initially identified as proteins upregulated in plant cells under heat stress, it is now widely recognized that their expression also increases in response to other abiotic stress, such as heavy metal stress, drought, cold and UV radiation (Jacob et al. 2017). These stress factors induce changes in the physiological, cellular and metabolic function of the cell, leading to the aggregation, misfolding and dysfunction of native and non-native proteins (Mishra et al. 2018). HSPs function as molecular chaperones and perform several protective roles that safeguard the cell from the harmful effects of stress. For example, they buffer and bind to the hydrophobic regions of unfolded polypeptides during translation, preventing aggregation and amino-terminal misfolding and ensuring proper folding of the polypeptide chain (Roy et al. 2019). Additionally, they assist in stabilizing protein structure, maintaining normal conformation, and regulating cellular homeostasis (Khan et al. 2021). Using SDS-PAGE, isoelectric focusing (IEF), western blot, and dot blot techniques, Polenta et al. (2020) discovered an increased expression of HSP in tomatoes in response to extreme heat and cold conditions. Their findings highlight the importance and application of HSP as a plant stress biomarker (Polenta et al. 2020). Table 7 highlights some examples of HSP expression and their mechanism of conferring tolerance to plants under different abiotic stress conditions.

**Table 7:** Heat shock protein as a protein biomarker in plants' adaptive response to various abiotic stress

Stress	Plant	Analytical Technique	Family/loci	Mechanisms/process	References
Heat	*Vitis vinifera* L.	RNA-seq and qRT-PCR	Family: sHSP LOC100263791	Accumulation of HSP prevents the damaging effects of heat stress by regulating the folding/ unfolding of cellular proteins and proteolytic degradation of proteins	Liu et al. (2012)
Heat	*Lycopersicon esculentum* cv*.* Cardenal	SDS Page, IEF and western blot	Family: HSP70 Locus: ABW76421 Family: sHSP Locus: er-sHSP	The overexpression of HSP improves heat stress tolerance by regulating the movement of proteins across the membrane and facilitating the refolding of denatured proteins	Polenta et al. (2020)
Drought	*Oryza sativa* L.	qRT-PCR	Family: HSP 81-1 LOC4345951	The elevation of the HSP gene expression enhances drought tolerance by preventing cellular proteins from denaturation	Verma et al. (2022)
Heat, drought, salinity, and heavy metal (cadmium)	*Hordeum vulgare* L.	qRT-PCR	Family: HSP70 LOC123453062 Family: HSP90 LOC123406222	Increased HSP expression promotes stress tolerance by regulating various biological activities, such as stabilizing macromolecular structures, controlling cell signalling, and influencing plant growth	Chaudhary et al. (2019)
Ultraviolet radiation	*Arachis hypogaea* L.	SDS-PAGE and MALDI-TOF	Not specified	Increased expression of HSP enhanced tolerance to UV radiation	Du et al. (2014)
Drought	*Pisum sativum* L.	SDS-PAGE, Western blot and qRT-PCR	Family: HSP22 LOC127075470	An increased cellular concentration of HSP confers tolerance by binding to proteins to prevent aggregation and protect the plasma membrane structure	Avelange-Macherel et al. (2015)
Heat and Drought	*Triticum aestivum* L.	SDS-PAGE	Family: sHSP LOC123124987 Family: HSP70 LOC100415839	Upregulation of HSP enhances stress tolerance by ensuring proteins are in their structural conformations and inducing the degradation of harmful polypeptides	Grigorova et al. (2011)
Heavy metal (Cu, Ni, Pb, and Zn)	*Zea mays* L.	SDS-PAGE and immunoblotting	Family: sHSP LOC100283886	An Increased cellular concentration of HSP protects the photosynthetic pigments from damages caused by heavy metal stress	Heckathorn et al. (2004)

As observed in abiotic stress, HSPs are also integral components of the adaptive strategies employed by plants to mitigate the adverse effects of biotic stress. They enhance tolerance to biotic stress by regulating the stability and accumulation of various stress-responsive proteins, including pathogenesis-related (PR) proteins and antioxidant enzymes, thus detoxifying reactive oxygen species and preserving membrane stability. Numerous studies have documented the differential expression of heat shock proteins (HSP) in response to biotic stress. In a study conducted by Li et al. (2021), it was observed that the increased expression of HSP24 improved the resistance of grape berries (*Vitis vinifera* Cv ‘Kyoho’) against Botrytis cinerea infection. The researchers reported that this was due to the physical interaction of HSP24 with pathogenesis-related (PR) proteins, leading to the activation of the salicylic acid defense pathway against the fungi. Table 8 shows similar examples of HSP expression under different biotic stress conditions, including their mode of action.

**Table 8:** Heat shock protein as a protein biomarker in plants' adaptive response to various biotic stress

Stress	Plant	Analytical technique	Family/loci	Mechanisms/process	References
Fungal (*Golovinomyces orontii*)	*Helianthus annuus* L.	LC–MS/MS	Family: HSP70 LOC110940013	Accumulation of HSP enhances plants' immunity by inhibiting pathogen invasion and mycelium spread within the plants' tissue	Kallamadi et al. (2018)
Fungal (*Diaporthe caulivora*)	*Glycine max* L.	RNA-seq and qRT-PCR	Family: sHSP LOC100798298 Family: HSP 70 LOC100809773	Upregulation of HSP increased resilience to fungal infection by increasing the accumulation and stability of plant defense receptors such as pattern-recognition receptors (PRRs) and nucleotide-binding domain leucine-rich repeat-containing receptors (NLR)	Mena et al. (2023)
Fungal (*Plasmopara viticola*)	*Vitis vinifera* L.	SDS PAGE and liquid chromatography-mass spectrometry	Family:HSP70.2 LOCIRVW33792 Family:HSP90.6 LOCIXP_059599446	Upregulation of the HSP increases immunity against fungal invasion by regulating immunity signalling and inducing the synthesis of resistance proteins	Liu et al. (2021)
Nematode (*Heterodera glycines* Ichinohe)	*Phaseolus vulgaris* L.	RNA-seq and qRT-PCR	Family: sHSP PHAVU_002G231700g	Upregulation of HSP enhances tolerance to nematode attack by preventing degradation of antioxidant enzymes	Jain et al. (2016)

## Antioxidant enzymes as biochemical markers in plant stress responses

Enzymatic antioxidants are essential in promoting plant growth and development by counteracting the deteriorating effects of oxidative stress. They break down and eliminate free radicals produced in plant cells during biotic and abiotic stress (Saisanthosh et al. 2018). These antioxidant enzymes include superoxide dismutase (SOD), catalase (CAT), peroxidase (POX), ascorbate peroxidase (APX), glutathione peroxidase (GuPx), and glutathione reductase (GR). SOD catalyzes the conversion of superoxide radicals to O_2_ and H_2_O_2_, CAT converts two molecules of H_2_O_2_ into water and O_2_, and POX scavenges H_2_O_2_ within extracellular spaces. In addition, APX utilizes ascorbic acid to reduce H_2_O_2_ to water, GPX catalyzes the breakdown of H_2_O_2_ and GR catalyzes the conversion of oxidized glutathione (dimeric GSSG) to reduced glutathione (monomeric GSH) (Rajput et al. 2021; Kapoor et al. 2020).

Abiotic stress triggers physiological and metabolic changes such as stomatal closure, reduced CO_2_ availability, and disruption of photosynthetic enzymes and photosystems (Sachdev et al. 2021). These stress-induced changes causes the accumulation of free radicals and reactive oxygen species such as singlet oxygen, superoxide ion, and hydrogen peroxide in various plant tissues, leading to oxidative damage and cell death (Dumanović et al. 2021). In response to increased ROS production, plants upregulate the synthesis of antioxidant enzymes to scavenge and maintain cellular ROS homeostasis, thereby mitigating the adverse effects of abiotic stress (Huang et al. 2019). Numerous studies have shown that under various abiotic stressors, plants upregulate antioxidant enzymes. Table 9 highlights a few of these.

**Table 9:** Antioxidant enzymes as biochemical markers in plants’ adaptive response to various abiotic stress

Stress	Plant	Analytical Technique	Name of antioxidant	Mechanisms/process	References
Drought	*Solanum lycopersicum* L.	2D-Gel electrophoresis and MALDI-TOF MS	SOD, CAT, and APX	Upregulation of SOD prevents cell damage by converting superoxide anion to hydrogen peroxide, while CAT and APX convert hydrogen peroxide to water	Rai et al. (2021)
Cold	*Glycine max* L.	RNA-seq and qRT-PCR	SOD and POD	Overexpression of SOD and POD genes enhanced tolerance to cold stress by preventing malondialdehyde and hydrogen peroxide accumulation	Hussain et al. (2023)
Drought	*Glycine max* L.	Spectrophotometry and SDS-PAGE	APX, GR, GuPx, CAT	An increase in the cellular concentration of APX, GR, GuPx, and CAT in response to drought stress detoxifies ROS and enhances drought-stress tolerance in the affected plant	Mishra et al. (2021)
Salinity	*Oryza sativa* L.	Spectrophotometry assay	CAT, GuPX and APX	Upregulation of the major antioxidant enzymes (CAT, GuPX, and APX) protects plant cells from the detrimental effect of ROS by scavenging accumulated ROS	Kibria et al. (2017)
High temperature, drought	*Triticum aestivum* L.	SDS PAGE	CAT and POX	Increased expression of CAT and POX enzymes protects cellular integrity by timely scavenging and detoxifying ROS	Khan and Farzana (2014)
Heavy metal stress (Arsenic)	*Oryza sativa* L.	Spectrophotometry	SOD, CAT, APX, and POD	Accumulation of antioxidant enzymes confers arsenic stress tolerance by scavenging ROS and reducing oxidative stress	Pooam et al. (2023)

Biotic stressors, such as pathogenic infections and wounding, trigger specific plant defence responses. This response involves generating elevated levels of reactive oxygen species (ROS), also known as oxidative burst, to prevent pathogen invasion and proliferation and facilitate death (Ali et al. 2018). ROS speeds up cell regeneration and wound healing by preventing pathogen invasion at the injury site (Polaka et al. 2022). Furthermore, ROS acts as a signalling molecule and regulates several signalling pathways involving cell wall modification, changes in gene expression and hypersensitive response (HR), further protecting plants from biotic stress (Lehmann et al. 2015).

Nevertheless, excess production of ROS beyond a specific concentration threshold disrupts cellular homeostasis, resulting in protein peroxidation, enzyme inhibition, breakdown of cellular components, DNA fragmentation, activation of apoptosis, and cell death (Wang et al. 2019b). Plants deploy antioxidant enzymes as the foremost defense line to counteract these detrimental effects. These enzymes play a crucial role in protecting plants from the harmful consequences of ROS generated by biotic stress factors (Sahu et al. 2022). Table 10 highlights examples of enzymatic antioxidants expressed in response to biotic stress factors and their mechanism of action.

**Table 10:** Antioxidant enzymes as biochemical markers in plants' adaptive response to various biotic stress

Stress	Plant	Analytical Technique	Name of antioxidant	Mechanisms/process	References
Fungi (*Fusarium oxysporum* f.sp. *ciceris* Foc)	*Cicer arietinum* L.	SDS-PAGE and Western blot	SOD, CAT, GR, APX, and GuPX	An Increased cellular concentration of SOD, CAT, GR, APX, and GuPX enhanced resistance against the pathogen by preventing lipid peroxidation and detoxifying ROS	García-Limones et al. (2002)
Insect (*Sesamia inferens*)	*Zea mays* L.	Spectrophotometry	CAT	Increased CAT activity enhanced resistance against insect attack by increasing cell wall resistance and activating defense genes	Sau et al. (2022)
Nematode (*Meloidogyne* spp)	*Ipomoea batatas* L.	Spectrophotometry	SOD, CAT and POD	Upregulation of the antioxidant enzymes enhanced stress tolerance by scavenging ROS	Yang et al. (2023a)
Bacteria *(Xanthomonas hortorum* pv. Pelargonii)	*Vigna radiata* L.	RT-PCR	SOD, APX, POX, and CAT	Increased expression of antioxidant enzymes modulates cellular ROS homeostasis by detoxifying excess ROS	Farahani and Taghavi (2016)
Fungi (*Colletotrichum gloeosporioides*)	*Vitis labruscana* L.	qPCR and spectrophotometry	CAT and SOD	Accumulation of the antioxidant enzymes increases tolerance against the pathogen infection,	You et al. (2022)

## Small RNA as a molecular biomarker in plant stress responses

Plant Small RNAs (sRNA) constitute a category of non-coding ribonucleic acid molecules spanning from 21 to 24 nucleotides in length (Morgado and Johannes 2019). They are ubiquitously distributed across diverse cell types and tissues, actively participating in various biological processes, including plant reproduction, growth, and response to biotic and abiotic stressors (Zhan and Meyers 2023; González Plaza 2020). Plant small RNAs (sRNAs) can be classified into several categories based on their biogenesis, including microRNAs (miRNAs), piwi interacting RNAs (piRNAs), small interfering RNAs (siRNAs), small nuclear RNAs (snRNAs), and small nucleolar RNAs (snoRNAs) (Brant and Budak 2018). However, miRNAs and siRNAs are the most widely studied, primarily due to their pivotal roles in enhancing plant resilience against abiotic and biotic stress factors (Chen et al. 2018).

miRNAs and siRNAs are both generated from double-stranded RNAs (dsRNAs) in a series of downstream reactions involving RNA polymerase and Dicer-like (DCL) proteins (Mahto et al. 2020). However, while DCL1 trims miRNA, siRNA is generated from multiple pathways involving diverse exogenous and endogenous dsRNAs precursors by DCL2-4 proteins. The generated miRNAs and siRNAs are then incorporated into Argonaute (AGO) proteins to form the RNA-induced silencing complex (RISC). This complex regulates target genes at the transcription and post-transcriptional levels (Tang et al. 2021).

The mechanism of action of sRNA at target sites involves transcriptional and post-transcriptional gene silencing through DNA methylation, RNA slicing, histone modification, and translational repression (Tang et al. 2022; Patel et al. 2020). Additionally, they exhibit diverse regulatory patterns in response to varying stress conditions, with upregulation observed in positive regulators and downregulation in negative stress regulators (Sun et al. 2021a). While specific sRNAs are conserved, overseeing shared traits across plant species, others are specific to particular species. Both species-specific and conserved sRNAs play a pivotal role in plant stress responses and can be used as plant biomarkers (Jyothsna and Alagu 2022).

sRNA acts as a modulator in response to diverse abiotic stress conditions. They regulate the upregulation or downregulation of target genes in stress-associated pathways at both the transcriptional and post-transcriptional levels (Mondal et al. 2023). For instance, miRNA contributes to enhanced drought tolerance by regulating the expression of drought-responsive genes, transcription factors, and other biomolecules, including proline, dehydrin, and LEA proteins (Saroha et al. 2017). Furthermore, miRNA regulates salinity stress tolerance by modulating ion homeostasis and hormone signalling pathways (Banerjee et al. 2017). More examples of some sRNA identified in various crop plants under abiotic stress, their identification procedures, and their mode of action are highlighted in Table 11.

**Table 11:** sRNA as a molecular biomarker under biotic stress

Stress	Plant	Analytical Technique	Type of sRNA	Mechanisms/process	References
Cold	*Solanum lycopersicum* L.	qRT-PCR	miR162	miRNA162 activated the ABA signalling pathway via CL1 cleavage, subsequently enhancing cold tolerance by regulating stomatal conductance and photosynthesis	Li et al. (2023)
Cold	*Citrus limon* cv. Eureka	qRT-PCR	miR396b	Upregulation of miR396 enhances cold tolerance by repressing the synthesis of 1-aminocyclopropane-1-carboxylic acid oxidase (*ACO*), the rate-limiting enzyme in ethylene synthesis, thus regulating ethylene-polyamine homeostasis	Zhang et al. (2016)
Heavy metal stress (chromium)	*Zea mays* L.	sRNA-seq and qRT-PCR	miRNA	Downregulation of maize miRNA improved stress tolerance by triggering the expression of stress-resistance genes, including ABC transporter G family member 29, transcription factors (TFs), Cytochrome P450, and superoxide dismutase	Adhikari et al. (2023)
Drought and heat	*Arachis hypogaea* L.	sRNA-seq and sRNA-blot	tasiRNA and miRNA	Accumulation of snRNA in plant tissue leads to the upregulation of stress-resilience genes, which increases tolerance to drought and heat stress	Mittal et al. (2023)
Salinity	*Oryza sativa* L.	sRNA-seq and Northern blot	miRNA	Upregulation of miRNA enhanced drought tolerance by inducing the activity of some TF such as NAC and AP2/EREBP and L-ascorbate oxidase	Parmar et al. (2020)

In addition, small RNA (sRNA) enhances plant tolerance to biotic stress by targeting genes involved in regulating multiple plant immune responses, including pathogen-associated molecular pattern(PAMP)- triggered immunity (PTI) and effector-triggered immunity (ETI) (Brant and Budak 2018). Both PTI and ETI work synergistically to induce various defense mechanisms, such as the accumulation of salicylic acid (SA), callose deposition on the cell wall, hypersensitive response (HR), generation of reactive oxygen species, expression of pathogenesis-related (PR) genes, and cell death at the infection site (Tang et al. 2021). For example, In barley (*Hordeum vulgare L.*), miRNA was upregulated by the infection of *Blumeria graminis* f. sp. *hordei*, a fungus that causes powdery mildew disease. Further experiments suggest that increased miRNA expression confers tolerance by activating a cascade of reactions, leading to disease resistance and cell death signalling (Liu et al. 2014). Table 12 highlights examples of some sRNA identified in various crop plants in response to biotic stress, their identification procedures, and their mode of action.

**Table 12:** sRNA as a molecular biomarker under biotic stress

Stress	Plant	Analytical Technique	Type of sRNA	Mechanisms/process	References
Nematode (*Heterodera glycines*)	*Glycine max* L.	qRT-PCR	phasiRNAs and nat-siRNA	The downregulation of siRNA induced modifications of target genes responsible for defense mechanisms, notably the ARF genes, ultimately resulting in increased tolerance to nematode attacks	Lei et al. (2022b)
Bacteria (*Ralstonia solanacearum*)	*Solanum lycopersicum* L.	sRNA-seq and RT-PCR	miRNA	induction and repression of different miRNA families prevented bacteria attack by regulating signal transduction and promoting cell wall synthesis	Shi et al. (2023)
Insect (*Cylas formicarius*)	*Ipomoea batatas* L.	qRT-PCR	miR167	Upregulation of miR167 enhanced resistance to insect attack by regulating the induction of multiple defense mechanisms, including the expression of SPL-family transcription factors, secondary metabolite synthesis, and epidermal hair development	Lei et al. (2022a)
Virus (Maize iranian mosaic virus)	*Zea mays* L.	sRNA-seq and qRT-PCR	miR395, miR166 and miR156	Upregulation and downregulation of specific maize miRNAs enhanced pathogen resistance by modulating defense genes such as heat shock proteins 70, ubiquitin, and 26S proteasome	Ghorbani et al. (2022)
Fungal (*Puccinia striiformis f.sp. tritici*)	*Triticum aestivum* L.	sRNA-seq and qRT-PCR	siRNA and miRNA	Differential siRNA and miRNA expression increased resistance to fungal attack by regulating multiple defense mechanisms, including silencing fungal genes required for pathogenicity in host plants and activating TFs and antioxidant enzymes	Mueth and Hulbert (2022)

## Structure–activity relationship of biomarkers

Understanding how biomolecules are structured is essential in determining their role in plants under various stress conditions. This is referred to as structure–activity relationships (SAR), which explores the relationship between a molecule's biological activity and its three-dimensional (3D) structure. Understanding the structure and c functional groups of a plant stress biomarker aids in predicting physiological and biochemical function (Šamec et al. 2021). For example, aquaporin is a transmembrane protein with unique characteristics that allow it to transport water in plants during osmotic stress (Wang et al. 2020). Aquaporin comprises 6 segments of alpha-helical hydrophobic protein domains and several NPA (asparagine-proline-alanine). The presence of hydrophobic segments and the formation of hydrogen bonds between water molecules and polypeptide residues enables rapid water transport within plant tissues (Adeoye et al. 2021). Similar examples showcasing the structure–activity relationship of various biomarkers can be found in Table 13.

**Table 13:** Relationship between a biomarker’s biological activity and its three-dimensional (3D) structure

Biomarker	Structure–activity relationship	References
Abscisic acid	The presence of two double bonds conjugated to the carboxylic acid at the 2-cis and 4-trans positions significantly impacts its role in regulating stress tolerance and several developmental processes in plants	Lin et al. (2005), Cutler et al. (2010)
Dehydrin	The presence of a distinctive lysine-rich conserved region known as the K-segment can form an amphipathic helix and bind to macromolecules to prevent stress-induced damage The presence of numerous charged and polar amino such as Ser, Gln, Pro, Lys, Glu, Ala, and Gly confers Antioxidant and metal chelating properties	Smith and Graether (2022), Rorat (2006)
Transcription factor	A DNA-binding domain aids the transcription factors in binding specifically to the cis-acting element in the promoter region of stress-induced genes An activation domain triggers downstream reactions that lead to the activation or repression of the gene	Kimotho et al. (2019)
Superoxide dismutase	Metal ions (Cu, Zn, Mn, and Fe) between the two sub-units act as cofactors in SOD, enhancing its catalytic activity of scavenging toxic metabolites by donating electrons to ROS	Stephenie et al. (2020)
Ascorbate peroxidase	The enzyme contains amino acid residues that boost its activity, such as lysine, cysteine, and arginine, which form hydrogen bonds with ascorbate/substrate and histamine, which aids the cleavage of the oxygen–oxygen bond in hydrogen peroxide Iron in the heme prosthetic group increases the enzyme’s catalytic activity	Dąbrowska et al. (2007)
Catalase	The active site comprises a heme group with three amino acid residues: tyrosine at the proximal end, histidine, and asparagine at the distal end, all of which are crucial for its catalytic activity	Karakus (2020)

## Recent trends in the application of plant stress biomarkers

### Application of plant biomarkers in crop engineering

To achieve sustainable agriculture and produce enough food for the world’s growing population, effective strategies for dealing with extreme conditions such as temperature extremes, pathogen attacks, herbivores, drought, salinity, and heavy metal stress are required (López-Arredondo et al. 2015). To achieve this, it is important to understand the cellular, epigenetic, and molecular mechanisms that orchestrate plant response to various biotic and abiotic stressors. This will lay the groundwork for engineering crops with faster growth rates, higher yield, and productivity (Jiménez Bremont et al. 2013). Modern crop science research has been transformed by the understanding of omic technologies (metabolomics, genomics, proteomics, and transcriptomics), which allow for more robust studies on the primary metabolites, proteins, genes, and molecular networking pathways associated with plant responses to various abiotic and biotic stresses (Yuan et al. 2008). The findings of these studies have aided in the identification of biomarkers that confer resilience to an adverse stress factor, as well as in the introduction of these desired characteristics into model crops and various economically important crops such as barley, maize, wheat, and rice, among others (Yang et al. 2021). These biomarkers are critical in developing new crop varieties (transgenic crops) that are more resistant to adverse environmental conditions (Rodziewicz et al. 2014). Plant biomarkers are widely used in genetic crop engineering to create transgenic crops with higher yields under adverse abiotic and biotic conditions (Leetanasaksakul et al. 2022; Bakhsh and Hussain 2015). Transgenic crops have proven to be a complementary and effective alternative in modern agriculture, increasing yield by 22%, reducing pesticide use by 37%, and increasing profit by 68%. These crops are grown on approximately 180 million hectares worldwide (James 2014).

Researchers have discovered that overexpressing the barley dehydrin gene (HVA1) in wheat can lead to the development of transgenic wheat that is better adapted to salinity and drought stress. The transgenic wheat plants displayed improved membrane stability and reduced electrolyte leakage, according to research by (Habib et al. 2022). The NAC transcription factor has been identified as a critical TF that enhances the resilience of cowpeas (*Vigna unguiculata* L. Walp.) to a range of environmental stresses, including drought, heat, cold, and salinity. By overexpressing two native cowpea NAC genes (VuNAC1 and VuNAC2), significant improvements in tolerance to these stressors were achieved. The resulting transgenic plants displayed enhanced antioxidant activity, membrane integrity, water use efficiency, and Na + /K + balance. These improvements culminated in an estimated threefold increase in growth and yield, (Srivastava et al. 2023).

### Application of plant biomarkers in crop breeding

**Fig. 2:**
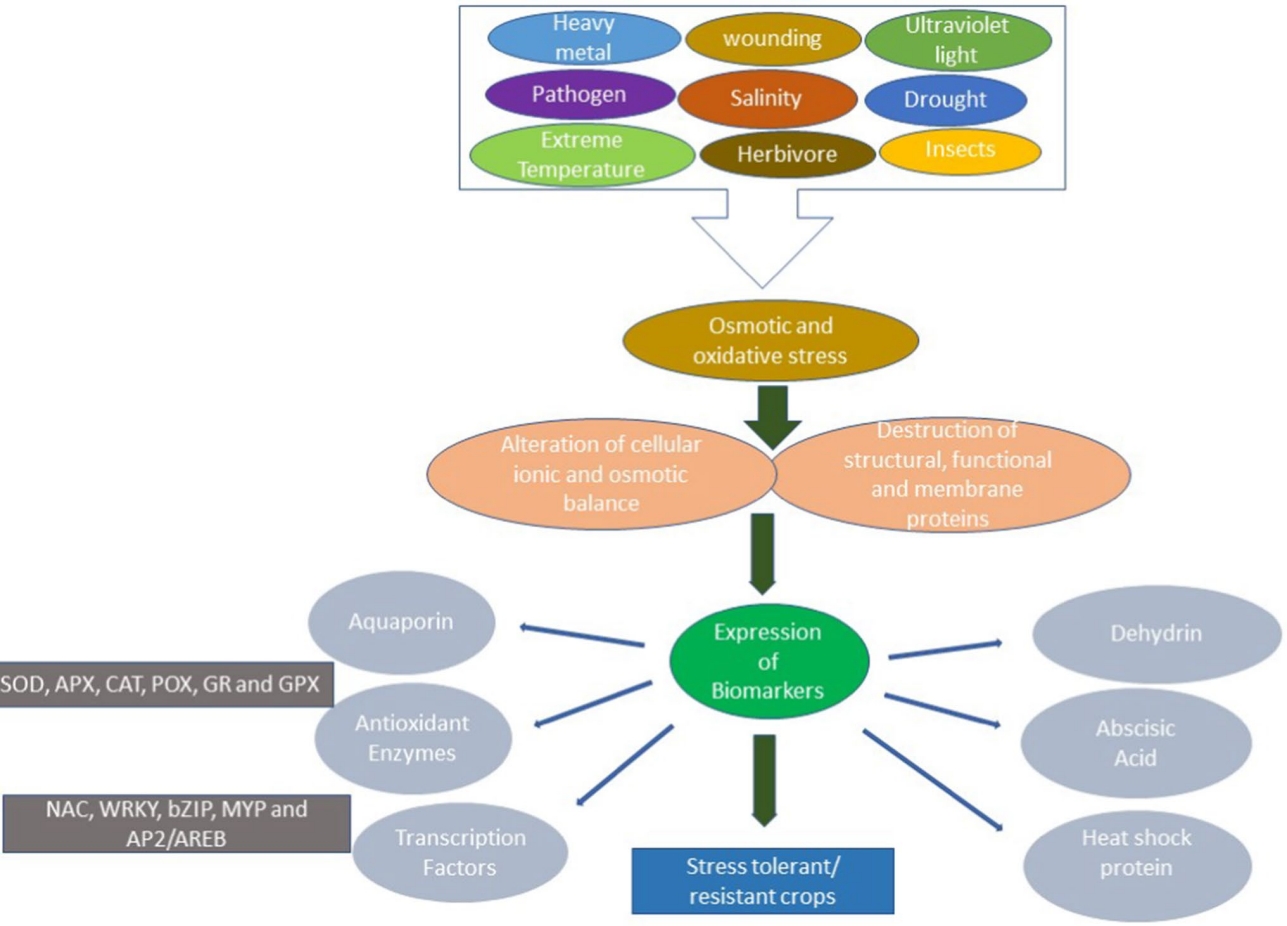
Schematic representation of plant response pattern to stress and expression of biomarkers (SOD—superoxide dismutase; CAT—catalase; APX—ascorbate peroxidase, POX—peroxidase; GPX—glutathione peroxidase and GR—glutathione reductase; HSP—heat shock proteins

When faced with challenging environmental or biological circumstances, plants may demonstrate alterations that involve either the activation or suppression of specific biomarkers, including transcription factors, enzymes, osmolytes, hormones, and small RNA (Isah 2019). These biomolecules are known to prevent the destruction of cellular components and restore homeostasis, which is critical for plant growth and development under stress conditions (Ben Rejeb et al. 2014). Figure 2 illustrates some biomarkers expressed by plants under stress conditions. Comparing the expression of these biomarkers provides information about the tolerance level of the plants and is also helpful for studying and analyzing different plant genotypes, species, and cultivars (Chaudhary et al. 2020). The knowledge of plant biomarkers has been used in crop breeding to identify phenotypes or cultivars that are more resilient or susceptible to different abiotic and biotic stress factors and has aided in selecting lines with better traits (Fahimirad and Ghorbanpour 2019; Dikobe et al. 2023). For instance, the molecular and physiological adaptations of different Andean potato genotypes (Tuberosum and Andigena) to drought were assessed by Vasquez-Robinet et al. (2008). The Andigena landraces accumulated more transcription factors, heat shock proteins, and antioxidant genes after 17 days of imposed drought, making them better adapted to drought than the Tuberosum genotype. The expressed biomarkers significantly classified the genotypes based on their tolerance and sensitivity level (Vasquez-Robinet et al. 2008). Similarly, Sathish et al. (2022) investigated the oxidative stress and antioxidant enzyme levels of 12 maize genotypes exposed to 8 days of severe drought stress. The cultivars responded differently to the imposed stress with varying concentrations of MDA and antioxidant enzymes. Based on the data obtained, three genotypes were classified as drought tolerant and others as drought sensitive (Sathish et al. 2022).

## Factors influencing the expression of plant biomarkers in crop plants under stress

Plant biomarkers are influenced by several factors including tissue or organ specificity, circadian readings, developmental stage, species, and cultivars, which cause differential expression patterns in crops exposed to the same stress conditions (Fernandez et al. 2016).

Under drought stress, two aquaporin proteins, PIPs and TIPs, were differentially expressed in *Brassica napus* plant tissues. PIPs and TIPs were downregulated in the root but upregulated in the leaves. Reduced AOP expression in the roots may be associated with the need to prevent water loss from the root due to water deficit. In contrast, increased leaf AQP expression enhances water transportation to regulate normal metabolic functions within the plant (Sonah et al. 2017). Similarly, Yu et al. (2019) observed variations in the expression of HSP in the leaves and roots of cassava plants exposed to drought stress. The leaves showed greater levels of upregulated HSP genes compared to the roots. Transcription factors are other biomarkers with variable expression patterns in different plant tissues. Using qRT-PCR, the expression profile of a specific wheat transcription factor, MYB4, in response to salinity stress, was investigated. The findings revealed increased expression levels in the shoots, whereas a simultaneous reduction in expression was observed in the roots (Sukumaran et al. 2023). Furthermore, the exposure of pepper plants (*Capsicum annuum* L.) to pathogenic infection resulted in variations in the expression profile of antioxidant enzymes in both the roots and leaves (Zheng et al. 2004).

Data from several research studies has shown that plant biomarkers are differentially expressed at different stages of plant growth when exposed to the same stress condition. Castañeda-Saucedo et al. (2014) reported differences in dehydrin accumulation at the seed filling and pod formation stages of common beans (*Phaseolus vulgaris* L.) subjected to drought stress. In a similar experiment, Samarah and colleagues observed a more significant dehydrin accumulation in soybean seeds (*Glycine max* L.) at the maturity stage compared to the developmental stage (Samarah et al. 2006). Furthermore, high-throughput techniques, such as gene profiling and RNA sequencing, were employed to examine the expression of the transcription factor (bZIP) in wheat (*Triticum aestium* L.) plants exposed to heat stress. The investigation revealed variations in expression across various developmental stages, with the highest expression level observed at day five post-anthesis (Agarwal et al. 2019). Ge and colleagues studied the expression of aquaporin at different stages of germination in *Brassica napus* plants subjected to cold, salinity and drought stress. The findings revealed an up-regulation of the AQP genes at the germination and early seedling stage but downregulated at the maturity stage (Ge et al. 2014).

The time of the day or circadian changes have also been reported to influence the expression of biomarkers in plants under stress conditions. For instance, the expression of Aquaporin (PIP) was upregulated at dawn and downregulated at dusk in different plants, which may be due to an increase in the rate of transpiration during the day (Hachez et al. 2012; Heinen et al. 2014; Ding et al. 2020). In a recent study conducted by Lu et al. (2021), it was discovered that while cold stress induces the activity of transcription factors (OsDREB1B and OsDREB1C) throughout the day, peak levels were observed during daytime as opposed to nighttime. Similarly, in peach plants (*Prunus persica* L.), cold stress leads to a more significant induction in the activity of dehydrins (DHN 1 and 3) in the morning (Artlip et al. 2013). In addition, transcriptomic analysis revealed that heat stress response genes such as HSP, antioxidant enzymes and specific TFs are more induced by heat stress in the morning and early afternoon than at other times of the day (Bonnot et al. 2021; Blair et al. 2019; Lai et al. 2012). Abscisic acid is another plant stress hormone controlled by circadian changes, with peak levels observed at specific times during the day in different crop plants (Hotta et al. 2013; Khan et al. 2010).

Cultivar or plant species is another factor influencing the expression of biomarkers subjected to the same stress conditions. Several examples of this have been documented in literature. Under salinity stress, two rice cultivars (Cotaxtla and Tres Ríos) exhibited different NAC transcription factor expression patterns (García-Morales et al. 2014). Variations in the expression profile of dehydrin between two grape species (*V. vinifera* and *V. yeshanensis*) in response to drought were also reported by Yang et al. (2012). While there was an upregulation of dehydrin genes in the *V. yeshanensis* species between 1 and 2 days post-drought imposition, a response was only observed in the other species between 2 and 3 days post-drought treatment. The results of the field survey conducted by Oliveria and colleagues revealed that differential expression of antioxidant enzymes was observed among ten different cultivars of cowpea (*Vigna unguiculata* L.) under nematode infestation (*Meloidogyne incognita*) (Oliveira et al. 2012). In addition, transcriptome analysis has revealed variations in the expression of the transcription factors (bHLH family, AP2-ERF, MYB and WRKY) in the cultivars of potato, tomato and spinach when subjected to pathogen attack (Bayoumi et al. 2021; Kandel et al. 2020; Upadhyay et al. 2016).

## Conclusion

Abiotic and biotic stressors pose severe challenges to global food security, rendering current crop yield insufficient to meet future global food demand. These stressors have been documented as significant contributors to a substantial decline in crop yields, affecting developed and developing nations. Therefore, we need to focus on developing innovative technologies and methods to produce stress-tolerant, widely-adapted, and high-yielding crops under various stress conditions. One way to achieve this is by understanding plant response patterns to external factors. When exposed to an environmental or biological stress factor, some biomolecules are up or downregulated in plants. Such biomolecules can function as effective plant biomarkers to monitor plant stress response. In this review, we discussed common biomarkers expressed by plants during abiotic and biotic stress conditions and the relationship between their biological activity and three-dimensional (3D) structures. Plant stress biomarkers have a wide range of applications in crop breeding and crop engineering in producing stress-resilient crops with higher yields under adverse conditions. In addition, they can be used for identifying plant cultivars, species, and genotypes with the desired or improved traits. Considering the potential of plant stress biomarkers, more research on the mechanism underlining plant responses to various stress factors and intensive development of analytical platforms and databases are encouraged to standardize plant stress biomarkers for use in breeding novel stress-resistant crop varieties with better yield.

## Data Availability

Not applicable.
